# Early recognition of speech

**DOI:** 10.1002/wcs.1213

**Published:** 2012-12-20

**Authors:** Robert E Remez, Emily F Thomas

**Affiliations:** Department of Psychology and Program in Neuroscience & Behavior, Barnard College, Columbia UniversityNew York, NY, USA

## Abstract

Classic research on the perception of speech sought to identify minimal acoustic correlates of each consonant and vowel. In explaining perception, this view designated momentary components of an acoustic spectrum as cues to the recognition of elementary phonemes. This conceptualization of speech perception is untenable given the findings of phonetic sensitivity to modulation independent of the acoustic and auditory form of the carrier. The empirical key is provided by studies of the perceptual organization of speech, a low-level integrative function that finds and follows the sensory effects of speech amid concurrent events. These projects have shown that the perceptual organization of speech is keyed to modulation; fast; unlearned; nonsymbolic; indifferent to short-term auditory properties; and organization requires attention. The ineluctably multisensory nature of speech perception also imposes conditions that distinguish language among cognitive systems. *WIREs Cogn Sci* 2013, 4:213–223. doi: 10.1002/wcs.1213

## INTRODUCTION

To understand an utterance, a perceiver must grasp its linguistic properties, the words, phrases and clauses, as well as the entailments of prior and looming events, spoken or not. Ordinarily, the attention of a perceiver is focused on a talker's immediate meaning, but the linguistic constituents that represent meaning are arrayed at the fine grain by linguistically governed segments, the consonants and vowels that compose each utterance. So, to recognize a sequence of spoken words necessitates the perceptual resolution of phoneme contrasts, a small set of characteristic distinctions that indexes words by their form. Listening to English, for example, is a version of this challenge that is well bounded, for there are only a dozen and a half contrast features and fewer than four dozen phoneme segments. In practice, the speech encountered by a perceiver exhibits vastly greater variation. In small part, this is due to the coarticulation of each phoneme with preceding and following phonemes, conferring the influence of receding and anticipated contrasts on motor expression and the consequent acoustic and sensory effects. Although phoneme contrasts contribute to the structure of an utterance, talkers simply cannot produce a standard set of acoustic properties, due to differences in anatomy. This variation in scale affects articulation and the resulting properties of speech as a consequence of the physics of acoustic resonance.

Anatomical differences aside, no talker simply speaks phonemes. An utterance expresses personal characteristics: a talker's maturity, sex and gender, vitality, regional linguistic habits, and idiosyncrasies of diction, not to mention idiosyncrasies of dentition. In conversations, an utterance can be produced carefully or casually, and can express features of mood or motive, making the contribution of canonical phonemic form just one of the determinants of articulation. This convergence of causes takes the phoneme sequence that indexes the words and gives it personal and circumstantial shape. It is implausible to view these effects as a kind of normally distributed variation in the phonetics of consonants and vowels.^1^ Overall, the expressed form reflects this bottleneck in production, resulting in huge variation in the physical effects of any single consonant or vowel. For the perceiver to recognize words requires the phonemic grain, but this is available only as a compromise among linguistic structure, paralinguistic expression, and circumstance. Accordingly, the relation between the phoneme series of a word and the actual spoken expression is commonly described as a problem of invariance and variability, that is to say, invariance in a recognized phoneme contrast despite variability in its phonetic, paralinguistic, and situation-specific characteristics.

The perceptual recognition of speech is not simply a topic of auditory concern, although it has been primarily so. Research on auditory sensitivity, whether psychoacoustic or physiological, has produced a description of the sensory effects of speech sounds, beginning in the cochlea and spanning the auditory nucleus to the primary auditory areas in vertebrates. Although these projects show the preservation of the acoustic features of speech, it is acknowledged^2^ that they offer a model of hearing speech sounds, not of listening to spoken language. The portrait of speech sounds provided by this research stops far short of an account of the resolution of linguistic properties of speech, and offers a description of the mere survival of the raw structure of the speech spectrum as it propagates along the auditory pathway. Acknowledging that recognition by features is inadequate both theoretically and descriptively to meet the challenge of invariance and variability, such accounts present a description of the audibility of speech sounds, and do not offer an account of speech perception.

From this perspective, it is sobering to note the persistence of the disposition to explain speech perception normatively, as if the resolution of phonemic properties obscured by paralinguistic and circumstantial influences were accomplished by sensitivity to distributions of sensory elements.^3^,^4^ This perennial theory of first and last resort must be false, however, appealing it is in its simplicity. At the heart of the normative conceptualization is the premise that the perceptual essence of a phoneme can be found in momentary articulatory or acoustic characteristics of speech. However, the acoustic details of speech reflect linguistic properties shaped by the characteristics of unique individuals and communities. In consequence, a statistical description of acoustic cues—the physical constituents of speech and their auditory effects—adds nothing more than precision to a premise that conflates linguistic and personal causes of acoustic variation. The normative premise is hopeful but false. Apart from an argument in principle, empirical work shows this clearly, motivated by the problem of perceptual organization, a fundamental aspect of speech perception that establishes the conditions requisite for the analysis of linguistic properties.

## PERCEPTUAL ORGANIZATION

How does a listener find a speech stream in sensory activity? Commonly, the environments in which speech occurs are acoustically busy, with several sources of sound in addition to a spoken source. Indoors, a reverberant enclosure sustains echoes of speech that exhibit spectral and temporal patterns similar to the perceived utterance. In social gatherings, several talkers speak concurrently. This central phenomenon confronting the scientific description of speech perception was well framed by Cherry^5^ as the ability to find and to follow a familiar if unpredictably varying signal within an acoustically lively setting similar in spectrum and pattern. Most descriptions of speech perception only begin after the auditory effects of speech are isolated from concurrent sound sources. This is readily seen in theoretical discussions of speech perception (see Refs 6–10; others reviewed by Klatt^11^). Indeed, whether or not an account is avowedly modular, asserting that special functions are required for the perception of speech, most are tacitly modular, explaining speech perception as if speech were the only sound a listener ever encountered, and as if only one talker ever spoke at once.^12^ Although this designation is easily met in a laboratory, it is not an adequate description of the ordinary perceptual challenges that confront a listener. Perceptual organization is the fundamental function presumed by such accounts: The means by which sensory effects of a spoken source are bound and rendered fit to analyze.

Technical research has produced two differing conceptualizations of perceptual organization. In one, direct measures of the perceptual organization of speech have been used to motivate a description.^12^–^14^ These studies reveal the action of a perceptual function that is fast, unlearned, nonsymbolic, sensitive to coordinate variation within the spectrum, indifferent to the detailed grain of sensation, and requiring attention. Although characteristics of this description continue to emerge in new findings, it offers a clear contrast to the portrait of perceptual organization given in a generic auditory account.^15^,^16^ This account derives chiefly from studies of acoustic patterns composed by design rather than sounds produced by natural sources.^17^ It elaborates the grouping principles noted by the Gestalt psychologist Wertheimer 90 years ago and expanded by Bregman^15^ and colleagues as an obligatory and automatic mechanism for sorting primitive elements of auditory sensation. According to this view, as the sensory elements of an auditory sample are individually resolved, they are rapidly and automatically arranged in segregated streams. Each stream is composed of similar sensory elements, and each stream is taken as the effect of a source of sound, whether the dimension of similarity along which binding occurs is temporal or spectral. This notion, recently reviewed by Darwin,^16^ asserts that grouping by similarity is an optimization produced by long-term evolutionary pressure on the auditory system; and, the perceptual organization of speech reduces to principles established in psychoacoustic studies of similarity grouping and their physiological counterparts, chiefly of neural comodulation and synchronization observed in studies of low-level hearing (see Refs 18 and 19). These two accounts, one based on direct examination of utterances and another on the psychoacoustics of arbitrarily constructed sound patterns, offer contrasting claims about the perceptual organization of speech. It cannot be very surprising that an account based on direct study of speech offers a decent estimate of some functions that apply in the early sensory processing of this kind of signal. More surprising is that the general auditory account fares so poorly in explaining the perceptual organization of speech despite assertions of its physiological plausibility.

### Direct Investigations of Speech

Direct investigations of the perceptual organization of speech have included studies that examined the intimate as well as the boisterous cocktail party. In the intimate case, the organizational challenge to perception is to take the physically varied and intricately patterned acoustic effects of the speech of a single individual and to group these into a perceptual stream attributed to one source of sound. The boisterous counterpart is a competitive organizational challenge, namely, to isolate the speech stream of a single individual from the speech of other talkers, reverberation, and sounds produced by nonvocal events.

#### Modulation Sensitivity

**FIGURE 1:**
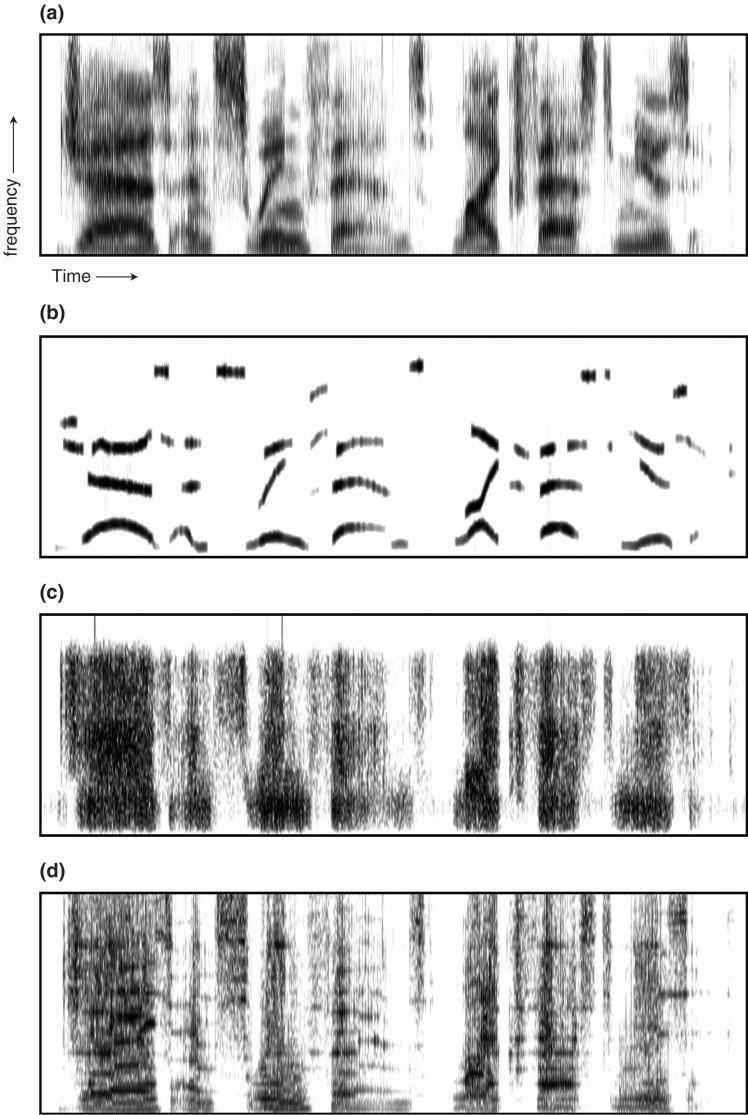
Spectrographic representation of four variants of the sentence, ‘Jazz and swing fans like fast music’. (a) Natural speech; (b) sine-wave speech; (c) noiseband vocoded speech; and (d) speech–music chimera.

In virtue of the indifference to short-term elementary acoustic characteristics, the organization of speech seems to depend instead on sensitivity to modulation directly. This has been observed in four independent cases: when time-varying sinusoids replicate the amplitude peaks of the center-frequencies of natural vocal resonances,^20^ when vocoded synthesis of speech in 1 kHz wide bands employs a carrier that is uniformly noisy^13^ or uniformly harmonic,^21^ and when the composition of the carrier varies arbitrarily,^14^ for instance, when the modulation characteristic of speech is imposed on a sample of sound produced by a collection of musical instruments (Figure 1).

The finding that organization does not depend on the resolution and categorization of individual acoustic moments was actually anticipated by the observation that the constituents distributed through a speech spectrum are not unique to speech. Each loses its phonetic effect when excised from a speech signal and presented alone.^22^ An individual acoustic moment in a speech spectrum can be grouped with the physically diverse and discontinuous set of natural vocal products that issues from a talker—the whistles, clicks, hisses, buzzes, and hums that speech is made of—because its relation to the ongoing acoustic pattern is consistent with a dynamically deformable vocal origin. A corollary of this claim is that the perceptually critical attributes of the speech stream are its modulations, and not the individual vocal postures, nor moments of articulatory occlusion and release, nor the specific acoustic elements that these brief vocal acts produce. This indifference of organizational functions to the short-term spectrum of speech demotes the classically designated acoustic cue to a role of diminished importance in perception.

Research on modulation sensitivity in the auditory system in the past decade produced preliminary descriptions both of the physical modulation of speech spectra^23^ and of the modulation-contingent trigger features of neural populations.^24^ Although these projects have observed the capacity of auditory neurons from the thalamus to the cortex to follow spectral modulations in speech, overall the findings describe sensitivity to a broader range of acoustic properties than is typical of speech, chiefly at the rapidly changing end of spectrum modulation. Further, no study has sought to identify neural populations that are effective for an auditory contour that is criterial for speech. One significant obstacle to identifying this aspect of modulation sensitivity in any nonhuman auditory system is that there is little behavioral evidence in animals of integration of the acoustically diverse speech stream. Proof that a speech sample introduced to the auditory pathway of a ferret or a cat evokes consistent neural effects shows that its acoustic constituents are audible, but is not evidence of integration of diverse acoustic constituents into a perceptual stream, neither by sensitivity to modulation nor by other means. A recent study of spoken word recognition by a chimpanzee^25^ presents a single potential instance of a nonhuman animal sensitive to modulation independent of the characteristics of the carrier. If adequate control methods were to prove eventually that perceptual integration and spoken word identification do occur in this nonhuman instance, on ethical grounds the auditory characteristics of this chimp are no likelier to be calibrated physiologically than those of a human listener. The neural correlates of modulation sensitivity in the perceptual organization of speech might be identifiable grossly in humans,^26^ but the search for neural circuitry responsible for binding diverse components based on their characteristic modulation must wait for research methods to catch up to the question.

#### Fast Pace of Integration

The physical modulations of speech correspond to the linguistic constituents of an utterance. Because linguistic constituents are nested, speech exhibits acoustic modulation ranging from fine to coarse temporal grain. At the slower end of this range, a breath group is typically 2 s in duration, and a clause within it about half as long. A phrase might last 500 ms, a syllable 200 ms, and a diphone—the concurrent production of a consonant and vowel—about 100 ms. It can be difficult to isolate the acoustic effects of individual segments, yet an estimate of 50 ms can be found, nonetheless, in the technical literature. Although each of these linguistic constituents must be resolved auditorily to apprehend the symbolic properties of speech, the psychoacoustic dynamic that applies is tied to the rapid fading of any auditory trace of sound. In this respect speech enjoys no special sensory status, persisting in its auditory form for 100 ms. Relative to almost every other kind of acoustic pattern, speech is familiar, which fosters a listener's proficiency in recasting a fading trace into a more durable form. The rapid pace of sensory integration is set by this psychoacoustic limit, and a window of integration 100 ms wide is fast in comparison to the leisurely pace of entire utterances. Claims of fast integration based on acoustic analyses of speech^27^,^28^ are corroborated by a diverse set of direct measures.^29^–^34^ Although some reports favor a description of sensitivity at the leisurely pace of syllables^35^–^37^ these findings are not consistent with the direct measures of performance, and therefore probably reflect integrative functions applied post-perceptually to linguistic properties already derived.^38^ The accretion of constituents superordinate to a segmental grain is an organizational function, but at this slow pace the ingredients to be integrated must already be resolved within a speech stream, much as a speech stream itself must already be resolved within an auditory scene to yield a coherent sensory sample fit to analyze.

#### Unlearned Sensitivity

Developmentally, the functions of perceptual organization are apparently unlearned, neither requiring nor admitting exposure to speech. In this respect, the grammatical functions in language, which require exposure to take shape, can be considered dissimilar from the perceptual functions that find and follow the sensory effects of speech. The youngest infants are attracted to speech before exhibiting sensitivity to linguistic structure.^39^ Indeed, an infant's attention to the linguistic properties of speech rests on a developmentally prior perceptual facility to organize the sensory effects of utterances. This necessarily precedes mastery of the native language, whether the coarse grain of prosody or the fine grain of phonotactics is concerned. An infant (at 14 weeks^40^; at 20 weeks^41^) demonstrates adult-like modulation sensitivity to spatially and spectrally disaggregated acoustic constituents of the rapidly changing consonant onset of a speech signal^40^ despite evident indifference to phonemic properties of the native language. An infant at this age is capable of finding speech in the nursery, yet it is unable to distinguish utterances on linguistically governed criteria, for instance, the phonotactics and prosodic attributes that distinguish the native language from utterances in as yet unencountered languages. There is some evidence that this acuity to the coherence of speech signals is especially protective in noisy environments during childhood^42^ and dissipates with age. Such a developmental course is consistent with an explanation of perceptual organization that is native, or very nearly so.

#### Nonsymbolic

Direct measures reveal that integration occurs preliminary to phonetic analysis, for which reason its character can be understood as nonsymbolic. These methods have used competitive listening conditions, in which the perceptual organization of speech is disrupted by an acoustic lure, depending on its pattern. An extraneous acoustic signal that masks a speech stream in whole or in part can block the perception of speech, but this form of competition, which simply obscures significant portions of the spectrum, is not especially informative about integrating functions. When both speech and lures are well above threshold and resolvable, it is possible to determine whether the pattern of the lure can compete for attention without also masking the speech. Indeed, a competitor is most effective when its frequency variation imitates a vocal resonance, although it need not be intelligible itself to compete for organization with acoustic components that are capable of forming an intelligible utterance.^43^,^44^ Ineffective competitors have exhibited natural amplitude variation in the absence of natural frequency variation, or frequency variation inconsistent with a vocal source. A dose–response effect was also reported in which the more speechlike the frequency variation of a competitor, the more it drew attention from the components of a speech signal.^45^ The property shared by the effective cases was frequency modulation typical of speech, despite remaining unintelligible, phonetically. Although amplitude co-modulation has figured prominently in auditory treatments of sensory integration, modulation at the pace of syllables is apparently ineffective in promoting the coherence of speech.

#### Indifference to Short-Term Acoustic Characteristics

Perhaps, the signature of the integrative challenge in the case of speech is the complexity and heterogeneity of the spectrotemporal pattern. Many natural sound sources exhibit nonlinear acoustic effects. Among these is the vocal apparatus, which is capable of issuing a wide range of acoustic properties. Modeling of acoustic phonetics^46^ has shown that small differences in configuration of the articulators can have large and discontinuous acoustic effects, producing spectrotemporal patterns in which resonances emerge and break up, for instance, when the release of an occluded tract produces a shock excitation, when narrowing produces frication, or when coupling of the oral and nasal pharynges produces discontinuous resonance and anti-resonance. Aperiodic effects are produced throughout the tract, from the larynx to the lips, and can be concentrated within narrow bands or spread across broad frequency ranges. Overall, speech is produced as a collection of different kinds of sounds rather than a single kind. Because the patterned spectrum of an utterance exhibits neither physical continuity, nor common modulation, nor a simple composite of similar acoustic elements, the organizational functions that find and follow speech must apprehend the coordinate if heterogeneous patterned variation. It is uncertain whether this is accomplished by a specific function that is reciprocal to the mechanics of vocalization. Speech is a well examined instance of mechanical sound production, yet no evidence suggests that the characteristics of the perceptual organization of speech must be unique among nonlinear sound sources. Whether these functions reflect a generic organizational function adequate for dynamically deformable sound sources, or a function unique to speech, can be answered by the relevant investigations, which have not yet occurred. Accordingly, there is little to report about the neural mechanisms that exhibit functional characteristics matched to these dynamic properties.

#### Requiring Attention

In ordinary listening, the sound of speech engages a listener's attention by virtue of its vocal timbre. Naïvely, this might seem to occur without effort or intention, but this ease is misleading. Studies that have used sine-wave speech to decouple vocal timbre from the effectiveness of spectral modulation reveal that perceptual organization is neither automatic nor obligatory (see Ref 47), contradicting the description of the perceptual module hypothetically dedicated to speech.^48^ The finding that the perceptual organization of speech requires attention also opposes the conceptualization of perception by means of modular pandemonium^49^ and its variants. These models share an assertion of passive organization and analysis. Descriptions of attention have rested on the notions of selection and dedication of specific cognitive resources. Alternatively, automatic cognitive functions occur neither with selection, nor with commitment of specific functional resources, nor with effort.

In the perceptual organization of natural speech, an impression of vocal timbre typically evokes attention to the time-varying spectrum as sound issuing from a spoken source, and a listener attends to the evolving sound pattern as speech. When the resonances are replaced with sinusoids, this creates an initial impression in a naïve listener of unrelated tones varying asynchronously in pitch and loudness. The tonal auditory impression of sine-wave speech is insufficient to summon attention to the pattern as speech. In this instance, the requisite attentional set can be supplied extrinsically. An instruction to listen to sine-wave replicas of spoken words as a kind of synthetic speech permits a listener to group the tones into an effective if anomalous vocal stream. Neural measures show that this condition—a spectral pattern adequately structured to evoke a phonetic impression in combination with attention—differed from contrasting cases in which the acoustic structure was inadequate, phonetically, or when the acoustic structure was effective but listeners did not attend to the incident pattern as speech.

### A Generic Auditory Account of Organization

Despite the evidence accumulating from direct studies of the perceptual organization of speech, a generic approach to auditory perceptual organization remains firmly established. At the heart of the conceptualization is the notion of similarity appropriated from Gestalt studies of visual and sometimes auditory grouping. In vision, the original demonstrations of grouping by similarity, or proximity, or closure, etc., relied on instances drafted with ink on paper. In audible examples of grouping, melodies were described as played on a musical instrument. More contemporary expressions of similarity grouping have invoked the motivation to understand the perceptual resolution of sound into streams, each issuing from an acoustic source in worldly scene. In contrast to the lofty ideal, the empirical development of the generic auditory account of perceptual organization has relied on arbitrary test patterns produced with oscillators and noise generators, largely eschewing the direct analysis of auditory scenes produced by actual mechanical sources of sound. As a consequence of these empirical practices, the generic auditory account includes an automatic mechanism that imposes an analysis of the auditory spectrum into its low-level features, and a grouping function that sorts features into segregated streams of similar features. Binding occurs across frequency to group simultaneously occurring features, and binding occurs over time to fuse features into temporal streams. Physiological evidence of some kinds of grouping have conferred biological plausibility on this account.

However, adequately this hypothetical explanation might fare in an ideal world of audiometric test patterns, an account devoted to similarity as the fundamental principle of grouping cannot be effective in explaining the perceptual coherence of a sound source as acoustically diverse as speech. Nonetheless, the generic account has been appealing because the hypothetical automaticity of its action and the simplicity of its grouping criteria seemed a good hypothetical fit to the low-level perceptual function of organization. Wholly apart from the claims of research on speech against the adequacy of this account, more specific counterevidence has undermined the description.

A serious challenge to the legitimacy of the generic auditory account of organization stems from a series of methodological investigations of the research paradigm used all but exclusively to establish the phenomena of grouping in human listeners. In these studies,^50^–^53^ the conventional method of inducing the formation of auditory groups was used, the cyclical repetition of a series of acoustic elements. For example, the perceptual evidence that organization tends to depend on the similarity in frequency among a set of tones is commonly observed in the subjective impressions of the order of a pattern of repeating tones. Evidence of perceptual organization of a single series of tones into two streams depended on the perceptual insensitivity to the intercalation order between streams, while within a stream of similar tones order was resolved veridically. These original findings led to a claim on general principle that grouping by similarity was automatic, fast and obligatory.

In contrast to the customary premise of the Gestalt-derived auditory account, recent projects found without exception that streams of reduplicated tones which formed along similarity criteria were slow to build. Converging measures exposed the fact that similarity-based streams were far from automatic in forming. When attention was drawn away from a stream that was already established, the perceptual state reset to an ungrouped form, indicating that cognitive effort is required to form streams even according to the similarity of arbitrarily composed elements. This disproved the long-standing claim that grouping by similarity is a primitive peripheral auditory mechanism acting rapidly, automatically, and effortlessly. Moreover, neurological patients exhibiting auditory spatial neglect did not establish auditory groups in the neglected hemifield. Overall, grouping failed to occur or was lost when attention was diverted from a tone stream by an audible or a visible distraction, or was simply unavailable due to neurological symptoms. In light of these reports, it is difficult to sustain the claim that perceptual organization includes a first step in which a fast, primitive, automatic function composes streams by auditory sensory similarity, whether for speech or other sounds. Direct performance measures of the perceptual organization of speech arguably offer a more realistic gauge of perceptual organization of complex sounds at this juncture.

### Multimodal Perceptual Organization of Speech

Studies revealed long ago that intelligibility improves relative to a unimodal case when a talker is both visible and audible. From the perspective of organization, the audiovisual circumstance has been conceptualized in two distinct ways. In one, perceptual organization applies within each modality, independently resolving the auditory and visual samples of the source of speech, and analysis of the linguistic properties proceeds to conclusion before sensory combination or alignment occurs. This conceptualization applies well to the familiar phenomenon of the McGurk effect.^54^ In that circumstance, a single syllable is presented for identification audiovisually. A limited response set promotes low uncertainty, and the experiment varies the extent of discrepancy, phonemically, between the visible and the audible display. Under such circumstances, perceivers report compromise between, for instance, a visually presented [ga] and an auditorily presented [ba], reporting [da]. Often, the syllable evoked in conditions of audiovisual discrepancy cannot be distinguished from a syllable perceived in consistent conditions. In some cases, incomplete compromise between the audible and visible rivals is reported. Sometimes, the conflict between sensory streams results in an impression that even violates the phonotactics of the language ([pta]). Impressions evoked in such presentations are both irresistible and primary, that is, impossible to attribute, subjectively, to rivalry between visible and audible experience of the spoken syllable. Because of this, some^55^ have claimed that this testing paradigm expressed the impenetrability to belief and indifference to sensory qualities that define a specialized perceptual module devoted to speech.

Other empirical phenomena of audiovisual speech perception have required a different conceptualization. In these reports, audiovisual integration is necessary for analysis, for which reason this condition is not amenable to an account of organization of each sensory stream in isolation from the other.^56^ To take an example,^57^ when audible properties of speech were taken from electroglottograph signals, these provided an impression of variation in vocal pitch, but when combined with a visible face producing fluent speech, the same auditory samples offer correlates of the voicing contrast and perhaps a bit of emphatically expressive laryngealization. Presumably, the visible face was intelligible on its own only intermittently, especially considering that the listeners were not expert speechreaders. Nonetheless, the combination was readily intelligible, evidence that intersensory combination was a condition on perceptual analysis, therefore, that primary organization necessarily included both senses.

When auditory and visual organization and analysis occur in parallel, the descriptive challenge of an account of intermodal organization is obviated. Unimodal organization is sufficient, because binding and phonetic perception occur independently in each modality.^58^ To explain the cases in which intermodal organization is requisite, although, the descriptive challenge is significant because of the incommensurate dimensionality of vision and hearing. Indeed, the only dimension common to visual and auditory samples is temporal, and empirical measures show clearly that temporal coincidence is neither necessary nor sufficient for intermodal binding to occur.^59^ This finding is a consistent observation whether the phenomenal objects are flashes of light and audible clicks, or speech. Although this research encourages a model of intermodal perceptual organization that includes a fast and nonsymbolic combination of sensory streams as a condition of phonetic analysis, neither the perceptual functions nor the functional neuroanatomy are well described.

## CONCLUSION

Most accounts of speech perception begin with an analysis of a speech stream. In this assumption, the hypotheses that follow must neglect the fundamental function of perceptual organization. Before perceptual analysis can resolve sensory properties into the symbolic entities on which recognition and comprehension depend, a prior function must find speech and follow its evolving sensory effects. In so doing, organization provides a coherent sensory sample appropriate to analyze. A complete description of the comprehension of spoken messages depends, therefore, on the resolution of a speech stream from its characteristic modulation. Although this function is apparently nonsymbolic in nature, without its action perceptual analysis can only apply indiscriminately to auditory samples, without disaggregating the sensory effects of speech and other sound sources.

The characterization given here has portrayed organization as prior and analysis as consequent, although this logical contrast belies the concurrency of these functions in practice. Organization proceeds at a rapid pace tied to the narrow window of integration; analysis projects auditory types into the symbolic properties that compose phonemes, syllables, words, and larger constituents by which meaning is conveyed. By this dovetailed relation of organization and analysis, the fragile auditory properties of speech become durable. Although a faded sensory sample of speech is lost to subsequent reorganization or reanalysis, the readily memorable results of linguistic analysis persist. Reimagining a talker's message must depend on this hardy representation of speech in the absence of a surviving trace of the original incident spectra.

What is the likely shape of an ultimate account of the early sensory functions in speech perception? The benchmarks from direct investigations of speech point to a perceptual resource that operates without much exposure, for it is evident in early infancy; it has a rapid pace, obliged by the decay of ephemeral sensory traces in a tenth of a second; it is widely tolerant of distortion, and apparently indifferent to the absence of short-term characteristics of natural vocalization; it requires attention, and is not characteristic of passive hearing; and, in the main, it is keyed to spectrotemporal modulation consistent with a deformable and nonlinear sound source. As with many accounts in contemporary research within cognitive neuroscience, the role of attention is a slumbering giant in this conceptualization. Many contemporary accounts of sensory functions and their cognitive effects presuppose attention, and portray a functional architecture within a perceiver's undifferentiated effort. New research has the potential to identify different types of attention and to establish principled grounds for the observation that a listener's belief determines whether a phonetically adequate sine-wave pattern is experienced as a set of contrapuntal tones or as an utterance. The contrast between hearing and listening also hinges on attention of some kind, and the validity of studies of passive exposure to speech, whether human or animal ears are enlisted, depends on an adequate account of attention no less than an account of the dimensions of audibility.

Sensitivity to complex spectrotemporal modulation also offers significant potential for investigation. Although psychoacoustics and electrophysiology identified sensitivity to simple forms of modulation long ago, the basis for integration across wide frequency spans typical in speech—at least 6 kHz—is not well understood. Neither is there a detailed account of rapid integration despite discontinuity and dissimilarity. Because this function does not require extensive exposure to language for its inception, it is doubtful that it reflects an aspect of perception that is dedicated to speech in a specific language. But, the singular focus of auditory research on sounds produced by linear emitters as a surrogate for mechanical sound sources denies us a generic account of perceptual organization of broadband, physically heterogeneous sound streams. Extending empirical practice to include natural sound sources might provide the principles underlying the perceptual organization of speech.

## FURTHER READING

Pardo JS, Remez RE. The perception of speech. In: Traxler M, Gernsbacher MA, eds. *The Handbook of Psycholinguistics*, 2nd ed. New York, NY: Academic Press; 2005, 201–248.

Remez RE, Rubin PE, Berns SM, Pardo JS, Lang JM. On the perceptual organization of speech. *Psychol Rev* 1994, 101:129–156.
